# Assessing Vocal Chanting as an Online Psychosocial Intervention

**DOI:** 10.3389/fpsyg.2021.647632

**Published:** 2021-06-01

**Authors:** Felicity Maria Simpson, Gemma Perry, William Forde Thompson

**Affiliations:** School of Psychological Sciences, Faculty of Medicine, Health, and Human Sciences, Macquarie University, Sydney, NSW, Australia

**Keywords:** music, chanting, COVID-19, music psychology, stress reduction, meditation, synchronization, relaxation

## Abstract

The ancient practice of chanting typically takes place within a community as a part of a live ceremony or ritual. Research suggests that chanting leads to improved mood, reduced stress, and increased wellbeing. During the global pandemic, many chanting practices were moved online in order to adhere to social distancing recommendations. However, it is unclear whether the benefits of live chanting occur when practiced in an online format. The present study assessed the effects of a 10-min online chanting session on stress, mood, and connectedness, carried out either in a group or individually. The study employed a 2 (chanting vs. control) × 2 (group vs. individual) between-subjects design. Participants (*N* = 117) were pseudo-randomly allocated across the four conditions. Before and after participation, individuals completed the Spielberg’s State Trait Anxiety Inventory, the Positive and Negative Affect Schedule, the Social Connectedness Scale and Aron’s Inclusion of Self in Other Scale. Online chanting led to a significant reduction in stress and an increase in positive affect when compared to the online control task. Participants who took part in group chanting also felt more connected to members of their chanting group than participants in the control group. However, feelings of general connectedness to all people remained similar across conditions. The investigation provides evidence that online chanting may be a useful psychosocial intervention, whether practiced individually or in a group.

## Introduction

Chanting is an ancient form of contemplative practice found in many cultures across the world. Still prevalent today, chanting is central to many traditions such as Yoga, Buddhism, Sufism, Shamanism, and Hinduism, where it is commonly practiced as a part of religious and healing ceremonies (Greene, 2016; Perry and Polito, 2021). Chanting is a focused attention meditative technique, where one directs attention toward sound, often referred to as a mantra, that is repeated either vocally or in the form of auditory imagery. It commonly accompanies spiritual rituals and may entail shared belief systems, but is also practiced in secular contexts and draws on general features of music such as rhythm, repetition, and collective vocalization (Lynch et al., 2018). Research suggests that participating in vocal chanting can lead to improvements in symptoms relating to stress, mood, anxiety, depression, and Post-Traumatic Stress Disorder (Wolf and Abell, 2003; Lutz et al., 2008; Bormann et al., 2014; Perry et al., 2016; Lynch et al., 2018). Moreover, chanting has been linked to social wellbeing, including increased social connection and altruism (Perry et al., 2016).

Despite the widespread practice of chanting rituals across cultures and traditions, there is remarkably little research on this pervasive form of musical behavior. Instead, the science and psychology of music has traditionally focused on the responses of Western listeners to Western tonal music (Thompson et al., 2019). However, there is growing interest in the psychological implications of chanting. Following increased awareness of biases in music teaching and scholarship, a trend has emerged to *decolonize* music curricula and scholarship, motivating researchers to expand and diversify the musical traditions and behaviors under investigation (Begum and Saini, 2019; Baker et al., 2020; Ewel, 2020). Moreover, research on chanting is especially significant for its unique mental and physical health implications, which have important applications during the global pandemic (Perry et al., 2021).

To date, there is little understanding of the unique properties of chanting responsible for such health benefits, but it is likely they arise from a convergence of multiple processes (Perry and Polito, 2021). Certain benefits of chanting may be unique to this particular practice–arising from processes associated with focused attention, vocal synchronization and shared goals–whereas other benefits may arise as a general consequence of music engagement. For example, music engagement across a range of contexts is associated with positive changes in emotional experience (Laird and Strout, 2007; Laird and Lacasse, 2014) and is often used for emotional self-regulation (Saarikallio, 2011; Bailey and Davidson, 2013; Juslin, 2019). Shared musical experiences have also been linked to improved ability to cope with adverse events (Von Lob et al., 2010) and improved mental health in clinical populations (Dingle et al., 2017).

**FIGURE 1:**
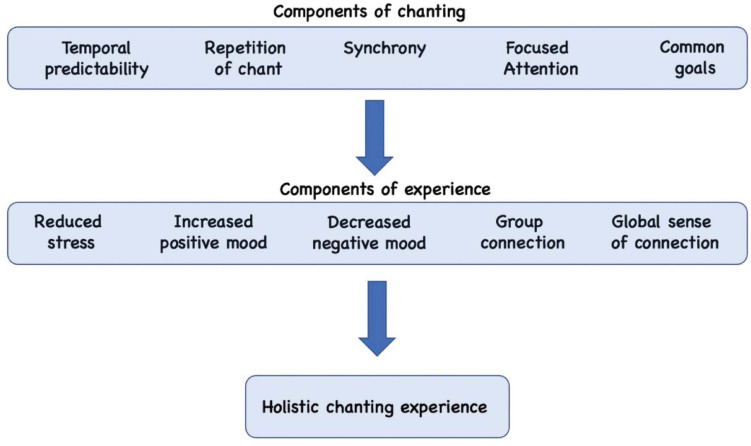
Various components of chanting converge to confer psychosocial benefits, including reduced stress, elevated mood, and increased feelings of social connection.

Figure 1 depicts some of the components of chanting practices that may confer psychosocial benefits. These properties include temporal predictability, repetition and synchronization of chanting (vocalized or imagined), attentional focus, and common goals. Common goals may include religious beliefs, attitudes or experiential goals shared by others in the chanting group, or others who practice chanting in the same tradition. In this framework, the various components of chanting combine to confer a range of psychosocial effects.

Whether such benefits are attenuated or otherwise changed for online chanting is an open question. Online chanting should engage the same psychological processes that are activated during live chanting, but those features and processes may be filtered and distorted by the medium of online technologies, such that potential benefits may well be diminished. For example, synchrony in vocalization may be hampered by the technical limitations of online technologies. Latency in sound processing can lead to variable delays in auditory feedback, resulting in imprecise timing of vocalization and group asynchrony. Similarly, common goals in group chanting (such as spiritual beliefs or shared attitudes) may be less tangible in remote, online contexts, leading to a reduction in a sense of solidarity with group members. Nonetheless, a number of documented benefits of chanting are expected to endure the transition from live to online conditions, as follows:

### Stress

The physiological demands of chanting may result in feelings of calmness and relaxation. Porges (2017) argued that the manipulation of breathing, coinciding with the recruitment of laryngeal nerves and pharyngeal nerves required for the vocalization in chanting, plays a critical role in the relaxation response–a response that has been linked to many contemplative practices (Benson et al., 1975). Chanting may decrease stress by encouraging cardiorespiratory synchronization, whereby breathing and heart rate become harmonized (Peng et al., 2004). The significance of these physiological mechanisms is supported by research on behaviors that make similar physical demands to those of chanting, such as singing, humming, and breathwork. Like chanting, such behaviors lead to a reduction of blood pressure, heart rate, and breathing rate (Lehrer and Gevirtz, 2014; Shaw et al., 2010). Bernardi et al. (2001) found that when participants recited a mantra “Om-mani-padme-om” or the prayer “Ave Maria” (Hail Mary), breathing rate in both circumstances dropped to six breaths per minute and became correlated with heart rhythm. The authors suggested that positive psychological outcomes could be attributed to this tempo (six breath per cycle) and alteration in respiration, with slower breathing rate promoting greater relaxation. Thus, the physiological demands of chanting may play a causal role in reducing stress, through increased parasympathetic activity and activation of the relaxation response. Such demands should be largely maintained in online chanting interventions.

### Mood

Research also suggests that chanting can lead to improvements in mood, and hence may be used as a tool for emotion-regulation. Perry et al. (2016) reported significant decreases in negative mood among both experienced and inexperienced chanters, regardless of whether participants were vocally or silently chanting the sound “Om” in a group. Improvements in positive mood were also observed, but only for inexperienced chanters who chanted vocally. In contrast, inexperienced chanters did not exhibit increased positive mood following silent chanting. Explicit vocalization of a chant may place higher demands on attention, allowing an individual to regulate their mood more effectively (Wolf and Abell, 2003). Thayer et al. (1994) noted that individuals use social engagement for emotion regulation, which may explain why group chanting or singing is particularly effective for improving mood. Thus, vocal group chanting may increase positive mood by stimulating attentional focus and promoting group solidarity. Online group chanting should also encourage attentional focus and group solidarity, but possibly to a more limited degree.

### Social Connection

Across cultures, chanting rituals are traditionally practiced in groups (Shearing, 2004; Perry et al., 2021). In general, group rituals are thought to enhance social bonding (Fischer and Xygalatas, 2014; Hobson et al., 2018), especially when individuals in the group become synchronized, as through music-supported movement (Wiltermuth and Heath, 2009; Kirschner and Tomasello, 2010; Bamford and Davidson, 2019). Increased positive mood has been linked to feelings of connection that arise when singing in a group (Clements-Cortés, 2015). Therefore, emotional benefits of chanting may be linked to the synchronous nature of singing in groups. Repetitive rhythms may allow individuals to optimize their attentional resources while facilitating tight synchronization (Keller et al., 2014; Margulis, 2014). Synchronization, a form of matching rhythmic behavior in time (Clayton et al., 2004), is associated with positive outcomes such as increased feelings of social connection, prosocial thoughts and behaviors (Valdesolo et al., 2010; Hobson et al., 2018). Perry et al. (2016) noted that group chanting increased feelings of altruism while enhancing positive mood and reducing negative mood. These findings are compatible with theories suggesting that social support can promote intrapersonal emotion regulation (Thayer et al., 1994). Similarly, stress-buffering models suggest that psychological stress can be reduced by feelings of social support (Dean and Lin, 1977; Cohen and Wills, 1985). It is currently unknown whether feelings of connection to others are enhanced by online chanting as they are with live chanting. Two features of live group chanting–interpersonal synchrony and shared goals–can be largely preserved in online chanting contexts, but may be limited in their efficacy. Variability in auditory feedback for online settings may restrict the degree of synchrony that can be achieved, and the remote nature of online chanting may make it difficult to appreciate any shared goals. Thus, online group chanting should give rise to a sense of connection with others, but possibly to a more limited degree than for live chanting contexts.

### Chanting as an Online Psychosocial Tool

Chanting is among the most universal musical practices worldwide, and can confer significant psychosocial benefits (Perry and Polito, 2021; Perry et al., 2021). During a global pandemic–with stress, depression and anxiety heightened–online practices that can reduce mental health problems and enhance wellbeing are of vital importance. To date, no study has examined the psychosocial consequences of online chanting. Is online chanting an effective tool for reducing stress, improving mood and social connection? Is the impact of online chanting greater when practiced in groups compared to individual chanting? Online wellness groups increased in popularity during the global pandemic, and have certain advantages over live wellness programs, including convenience, reduced costs, access, and safety from COVID infection. The presence of a group may provide motivation to take the process seriously, and other group members can provide insight, encouragement and emotional support. Such benefits of group participation may lead to increased feelings of social connection and belonging, which are associated with wellbeing (Holt-Lunstad et al., 2015).

**FIGURE 2:**
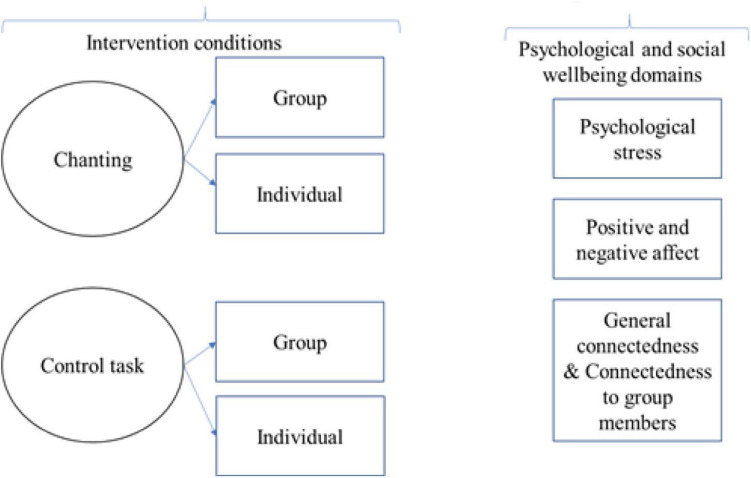
Illustration of the study design.

The goal of this investigation was to evaluate the impact of online chanting on stress, mood, and feelings of social connection. The conceptual framework for the design of the study is illustrated in Figure 2. Psychosocial measures were taken pre- and post-either chanting in a group or individually. To ensure that any effects observed arise from chanting, a control task was used in group and individual formats, and involved a 10-min listening exercise. The listening exercise required attention and engagement, but did not involve chanting. A 10-min intervention was chosen to detect the immediate effects of chanting, based on previous research in meditation and music that has also used 10–15 min interventions and detected significant effects (Lazar et al., 2000; Bhasin et al., 2013; Perry et al., 2021). Based on these previous findings, we predicted that chanting would decrease psychological stress, increase positive mood, decrease negative mood, and increase social connection. We also predicted that these effects would be greater when chanting in a group than when chanting individually.

## Materials and Methods

### Participants

117 participants completed the online study. Participants were recruited via two methods: the Macquarie University Psychology participant pool and social media. Those recruited through the Psychology Pool received two course credits in exchange for their participation. Those recruited through social media went into a draw to win one of three AUD$50 Amazon vouchers. Ethics for the experiment was approved by the Macquarie University Human Research Ethics Committee. Additional demographic information about these participants can be found in the “Results” section.

### Materials

Several measures were chosen to evaluate the impact of chanting on anxiety, mood, and feelings of connection, based on their internal consistency and effectiveness in revealing the effects of short interventions in relevant investigations, as follows.

#### Spielberger’s State Trait Anxiety

Psychological stress was measured via The State Trait Anxiety Scale (STAI; Spielberger et al., 1970). The STAI has been used to assess brief changes in stress and/or anxiety in a series of intervention studies (Thoma et al., 2015; Perry et al., 2016). This is a self-report questionnaire including 20 questions measuring state anxiety (present moment emotional states), and another 20 measuring trait anxiety (a more general, long-standing quality). For each item, participants rate an adjective on how well it represents their current level of stress. Responses for each item range from 1 (not at all) to 4 (very much so). For state anxiety, internal consistency was excellent at both time 1 (α = 0.91) and time 2 (α = 0.90). Similarly, trait anxiety (only take at time 1) was acceptable (α = 0.90).

#### Positive and Negative Affect Schedule

The Positive and Negative Affect Schedule (PANAS; Watson et al., 1988) is a 20-item self-report questionnaire used to assess Positive and Negative Affect. The PANAS has been used extensively to measure state-dependent changes in mood (Mitchell et al., 2009; Drake and Winner, 2012; Perry et al., 2016). Participants indicate how much they believe an adjective describes their current mood via a 5-point Likert Scale, ranging from 1 (very slightly or not at all) to 5 (extremely). The PANAS has an acceptable level of internal consistency ranging between 0.86 and 0.90 for positive affect and 0.84–0.87 for negative affect (Magyar-Moe, 2009). For the present study, the phrasing of the PANAS included “at the present time” to measure the individual’s present affect levels in response to the intervention, taken at time 1 and time 2. For positive affect internal consistency was excellent at both time 1 (α = 9.34) and time 2 (α = 0.96). For negative affect scores internal consistency was also at an acceptable level at time 1 (α = 0.88) and at time 2 (α = 0.91).

#### Group Connection: Aron’s Inclusion of Other in Self Scale

**FIGURE 3:**
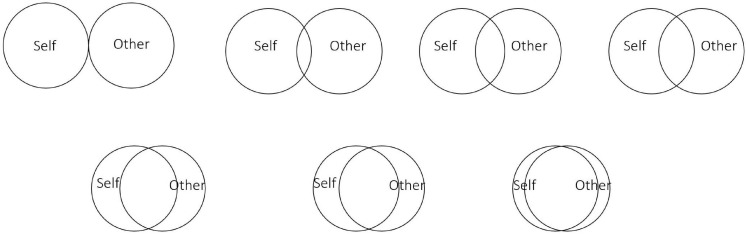
The Inclusion of Other in Self Scale illustrates feelings of unity between self and other (Aron et al., 1992).

The Inclusion of Other in Self Scale (IOS; Aron et al., 1992) is used to measure perceived connection to others. The IOS has been used in repeated-measures designs to assess explicit feelings of connection to other people, and changes in feelings of connectedness following interventions (Agnew et al., 2004; Vezzali et al., 2012; Charles et al., 2020, 2021). The questionnaire includes seven pairs of circles, or Venn diagrams as shown in Figure 3. Two circles with no overlap is indicative of no closeness to others, whereas one circle completely engulfed within the other circle represents extreme closeness. We used a variation of the IOS to ask individuals how close individuals felt to other members of the group using the phrase, *“Which circle best describes your relationship with others in your group?”*

#### General Connection: Social Connectedness Scale and IOS

To assess general connectedness, a composite measure with seven items was used. The first six items were from SCS (Pavey et al., 2011), a state-based scale, used to measure feelings of general social connectedness in previous meditation research (Aspy and Proeve, 2017). Six statements assess the state of social connection on a 7-point Likert scale ranging from 1 (strongly disagree) to 7 (strongly agree). For instance, participants are presented with statements such as “*At the present moment I feel a bond with other people*”. The IOS was used as the last item on the composite measure, however, participants were asked “*Which circle best describes your relationship with other people in general?*” The formation of the composite outcome variable was based on procedures used to form a composite variable in previous studies of the effect of rituals on mood and social bonding (Charles et al., 2020, 2021). The internal consistency for the combined measure was acceptable at both time 1 (α = 0.84) and time 2 (α = 0.87).

#### Previous Experience

To understand previous experience with chanting and meditation, participants were asked *“How often do you engage in meditation?* and “*How often do you engage in chanting?”* Participants responded on a Likert scale ranging from 1 (no experience) to 6 (daily practice).

#### Task Focus

To assess task engagement, participants were asked *“To what extent were you engaged in the task?”* and *“To what extent were you involved in mind wandering?”* Participants responded on a Likert scale ranging from 1 (none of the time) to 5 (all of the time).

#### Chanting Audio

The chanting condition involved the use of an audio recording of the sound “om” chanted in a male voice, taken from the YouTube link at: https://www.youtube.com/watch?v=yoYrLM5rGX8&list=RDyoYrLM5rGX8#t=22.

#### Control Audio

The control audio was a 10-min description about Yorkshire spoken in a female voice. This recording was chosen due to its previous use as a relaxation control task in meditation research (Lueke and Gibson, 2015). Moreover, the use of a relaxing listening condition should elicit a level of focused attention that is comparable to that required for chanting, thereby permitting the potential detection of an effect of the chanting itself.

### Procedure

**FIGURE 4:**
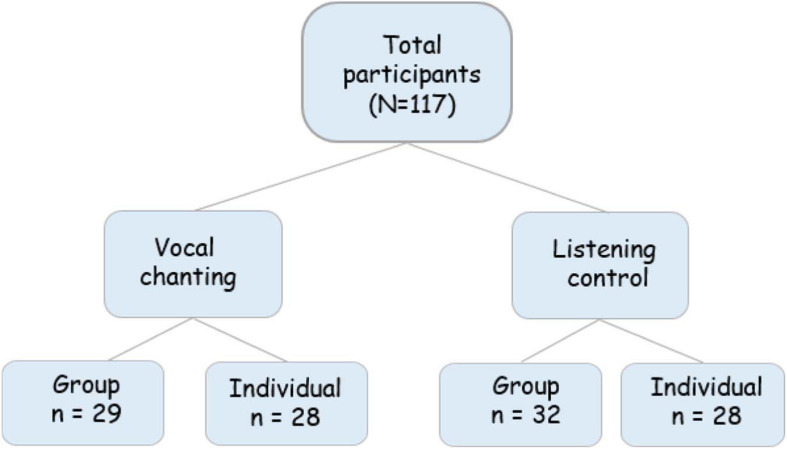
Illustration of the conditions and participants.

All participants were tested on Zoom, which is a free program for online video conferencing. Upon signing up, participants were emailed an explanation of the study and a Zoom link to the experiment. Participants were randomly allocated to one of four conditions: individual chanting; group chanting; individual control; group control. See Figure 4 for a visual representation of the four conditions. After entering the Zoom meeting, participants were given a brief introduction and sent a link to the Qualtrics survey. This survey included a consent form, demographics, PANAS, STAI, SCS, and IOS. Following this stage, the researcher provided instructions for the listening or chanting task, depending on the condition.

**FIGURE 5:**
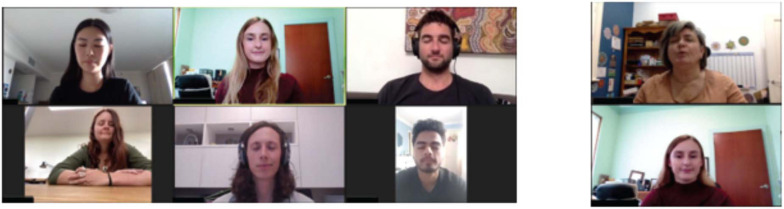
Example of group and individual conditions.

For both chanting and listening tasks, the experimenter placed the participants on mute, but their webcam remained on. For the chanting task, the researcher provided a demonstration of the chanting and instructed participants to chant the sound “Om” for 10 min with eyes closed. Participants in the group chanting condition were instructed to chant alongside the audio, while participants in the individual condition chanted without the audio. The audio was only used in the group condition to mimic the effects of synchronization when an individual chants with a group, as Zoom software is non-optimal for participants to hear each other while chanting. Participants were not given instructions on the length of each chant and number of times they were to repeat it during the 10 min. Although this approach may have given rise to differences in breathing rates between the vocal and listening conditions, it was adopted to ensure the ecological validity of the conditions. Specifically, the group condition simulated a group of individual chanting in synchrony with one another, whereas the individual condition simulated individuals chanting alone and therefore not attempting to synchronize with others. Figure 5 illustrates the online group and individual conditions (by permission). After 10 min, the researcher asked the participants to open their eyes and return to the Qualtrics survey. The post-intervention survey included the same items from the pre-intervention survey, without the STAI trait measure and with the addition of a manipulation check. Upon study completion participants were debriefed on the full research question and objectives.

## Results

### Descriptive Statistics

#### Participants

Forty-four females and 72 males between 18 and 65 years (*M* = 29.43, *SD* = 12.17) participated in the experiment. One participant was excluded because they did not complete the first half of the survey, so 117 participants were included in the data analyses, distributed among the four experimental conditions as displayed in Table 1. Religious traditions reported included 31.2% Spiritual, 25.6% Christian, 21.37% secular, 6% Yoga, 6% Hindu, and the remaining 21% included Islam, Buddhist, Jewish, and other religion.

**TABLE 1:** Standardized change scores across conditions (measured at pre-intervention to post-intervention; unadjusted).

Participant group			Measure			
	*–*	Stress	Positive affect	Negative affect	General connection	Group only connection
Group chant (n = 29)	*Z*	4.06	3.14	–2.03	0.20	0.55
Group control (n = 32)	*Z*	–0.59	–3.72	–1.06	–0.75	–0.031
Individual chant (n = 28)	*Z*	–4.07	1.32	–1.79	–0.93	
Individual control (n = 28)	*Z*	–0.28	–2.61	–2.32	1.32	

#### Inferential Statistics

Using Gpower software, it was determined that to detect a significant interaction between chanting style and social context, with power of 0.8 and alpha set at 0.05, a minimum of 28 participants were required for each group. First, ANOVA was used to assess effects of Intervention (chanting vs. control) and Group Size (individual or group) on stress, positive affect, negative affect, connectedness to the intervention group, and general connectedness. Change scores of dependent variables were used for these analyses. Standardized change scores observed for all measures and each condition are presented in Table 1 (for pre- and post-scores of all measures, See “Appendix”). Following the analyses, regression modeling was conducted to confirm that significant effects remained when a range of covariates and confounder variables were included in the model, and to evaluate the unique contribution of these variables to the outcomes.

##### Stress reduction

A two-way ANOVA revealed a main effect of Intervention, *F*(1, 113) = 16.71, *p* < 0.001, η*^2^* = 0.09. Participants in the chanting conditions demonstrated greater decreases in stress (*M* = −4.07, *SE* = 0.79), compared to participants in the control conditions (*M* = −0.45, *SE* = 0.77). There was no main effect of Group Size, *F*(1, 113) < 1.0, *ns*, and no interaction, *F*(1, 113) < 1.0, *ns*. That is, the benefits of chanting on subjective stress did not depend on whether participants chanted individually or in a group.

##### Positive affect

A two-way ANOVA revealed a main effect of Intervention on positive affect, *F*(1, 113) = 18.92, *p* < 0.001, η*^2^* = 0.14. Participants in the chanting condition reported greater increases in positive affect (*M* = 2.23, *SE* = 0.89), compared to participants in the control conditions (*M* = -3.16, *SE* = 0.87). There was no main effect for Group Size, *F* (1, 113) = 0.01, *p* = 0.933, and no interaction, *F*(1, 113) = 0.22, *p* = 0.637. That is, the benefits of chanting on for positive mood did not depend on whether participants chanted individually or in a group.

##### Negative affect

A two-way ANOVA revealed no main effect of Intervention on negative affect *F*(1, 113) < 1.0, *ns*. Similarly, there was no main effect of Group Size, *F*(1, 113) < 1.0, *ns* and no interaction, *F*(1, 113) < 1.0, ns.

##### Group connectedness

A one-way ANOVA (group interventions only) revealed an effect of Intervention on group connection, *F*(1, 59) = 6.34, *p* < 0.02, η*^2^* = 0.10. Participants in the chanting condition reported a greater increase of connection to their group members (*M* = 0.55, *SE* = 0.17) than participants in the control condition (*M* = −0.031, *SE* = 0.16).

##### General connectedness

A two-way ANOVA revealed no effect of Intervention on general connectedness, *F*(1, 113) = 1.01, *ns*, no effect of Group Size, *F*(1, 113) = 1.13, *ns*, and no interaction, *F*(1, 113) < 1.0, *ns*. Thus, although feelings of connection to immediate group members was higher for participants who chanted compared to those in the control condition, this effect did not extend to feelings of connection to all people.

#### Regression Modeling

To further explore the data, regression modeling was conducted. Based on a preliminary analysis of various predictors, a model of outcome measures was created with three predictors included in addition to the two independent variables representing our manipulations of the chanting intervention: trait anxiety, level of engagement, and prior chanting experience. Other predictors were considered (e.g., age, gender, and mind wandering) but their inclusion did not substantially alter the model outcome. Trait anxiety was included on the assumption that the benefits of chanting on mood, stress, and sense of connection may be more pronounced for individuals with high trait anxiety than individuals who are already comparatively relaxed. Level of engagement was included as a predictor because the benefits of chanting may be partly explained by the engaging nature of chanting relative to other activities. Indeed, ratings of engagement were significantly higher for participants in the chanting conditions (*M* = 4.44, *SE* = 0.13) than for those in control condition (*M* = 3.88, *SE* = 0.13), *F*(1, 115) = 9.40, *p* < 0.01, η*^2^* = 0.076. Prior chanting experience was included because the impact of chanting may differ for novices and experienced chanters. Another goal of the regression analysis was to confirm that the effects of chanting on stress reduction, positive mood, and group connection reported above remain when these three variables are controlled.

##### Stress reduction

Firstly, change in stress was modeled with the five predictors (*R* = 0.55, *F* = 9.77, *p* < 0.001). As in the ANOVA, the Intervention (chanting vs. control) remained a significant predictor (*B* = 2.11, *t* = 2.09, *p* < 0.04), and Group Size was non-significant (individual or group). That is, stress reduction was greater for participants who engaged in chanting than those who did not, and this effect was similar for participants who chanted individually or in a group. Trait anxiety was also a significant predictor in the model (*B* = −0.15, *t* = −3.38, *p* < 0.001), indicating that stress reduction following any intervention was greater for participants with high trait anxiety than for those with low trait anxiety. Engagement in the intervention was also a significant predictor (*B* = −2.01, *t* = −3.93, *p* < 0.001), indicating that individuals who were more engaged with the intervention experienced greater stress reduction than those who were less engaged. Finally, prior chanting experience was a significant predictor (*B* = −0.65, *t* = −2.05, *p* < 0.05), indicating that those with more chanting experience showed slightly more stress reduction than those with less chanting experience.

##### Positive affect

The change in positive affect was modeled with the five predictors; intervention, size, engagement, trait anxiety, and prior chanting experience (*R* = 0.44, *F* = 5.38, *p* < 0.001). As in the ANOVA, Intervention (chanting vs. control) remained a significant predictor (*B* = −4.639, *t* = 2.09, *p* = 0.039) whereas group size (group vs. individual) was not significant (*p* = 0.638). That is, an increase in positive affect was greater for participants who engaged in chanting than for participants who did not, and this effect was observed regardless of whether chanting was done in a group or individually. No other covariates (trait anxiety, engagement, or prior chanting experience) were significant predictors in the model.

##### Negative affect

The change in negative affect was modeled with the five predictors (*R* = 0.46, *F* = 6.04, *p* < 0.001). As per ANOVA, neither Intervention (chanting vs. control) nor Group Size (group vs. individual) were significant predictors. However, trait anxiety significantly predicted changes in negative affect (*B* = −0.195, *t* = −4.61, *p* < 0.001). Specifically, a greater reduction in negative affect was associated with higher levels of trait anxiety (regardless of condition). Similarly, engagement in the intervention (whether chanting or listening) was a significant predictor of the change in negative affect (*B* = −1.082, *t* = −2.24, *p* = 0.027). Those who reported a greater level of engagement experienced a greater reduction in negative affect.

##### Group connectedness

The change in connectedness to group members (for group conditions only) was modeled with four predictors (Group Size was omitted; *R* = 0.31 *F* = 1.57, *p* = 0.194). As in the ANOVA, Intervention (chanting vs. control) remained a significant predictor (*B* = −0.571, *t* = 2.30, *p* = 0.025). However, no other variables were significant predictors in the model.

##### General connectedness

The change in general social connectedness was modeled with the five predictors (*R* = 0.14, *F* = 0.80, ns). As per the ANOVA, neither Intervention (chanting vs. control) nor Group Size (group vs. individual) were significant variables in the model. No other variables were significant predictors in the regression model.

## Discussion

The present investigation aimed to examine whether chanting can be an effective online tool to improve psychological and social wellbeing, focusing on stress reduction, increased positive mood, decreased negative mood, and social connection. Chanting online resulted in a significant decrease in stress, with similar decreases observed for both group and individual chanting. Similarly, positive mood improved more for participants who chanted (whether individually or in a group) than for those who did not. Interestingly, negative affect was unaffected by the experimental manipulations. Lastly, participants in the group chanting condition reported higher levels of connection to their immediate group than those in the group control condition (who reported decreased connection to their group). However, chanting in a group did not result in changes to general feelings of connectedness to all people. The findings inform our framework for understanding the processes by which chanting gives rise to psychosocial benefits.

Figure 1 identified five aspects of chanting that may be relevant to such benefits: temporal predictability, repetition, synchrony, focused attention, and common goals. All of these attributes can be retained to some degree in an online intervention, with temporal predictability and repetition virtually unperturbed. However, certain aspects of chanting may be experienced in a weakened form in an online context. First, variable delays in auditory feedback among participants may result in poor synchronization among participants, reducing the potential of synchrony to confer psychosocial benefits. Second, participating remotely may afford greater potential for distraction given uncontrolled individual (home) conditions, reducing the degree and maintenance of focused attention during the intervention. Third, the common goals of chanting, which may be palpable when chanting in a live context, may be less evident or intangible when chanting as part of an online intervention, reducing the sense of solidarity and any cascading benefits that common goals would normally confer.

### Stress

Chanting led to significantly reduced levels of psychological stress. These results corroborate previous findings that vocal chanting can reduce psychological stress (Perry et al., 2016). Considering scientific paradigms of physiological stress and relaxation responses Porges (2017), it is reasonable to conclude that the manipulation of breathing, and therefore heart rate, was responsible for this decrease in psychological stress. The link between a physiological relaxation response and stress reduction has been demonstrated in contemplative practices (Benson et al., 1975; Bhasin et al., 2013), and slowed breathing and heart rate are associated with psychological benefits during religious chanting (Bernardi et al., 2001). The current results illustrate that such benefits are also observed following online chanting interventions.

It was predicted that when chanting was done in groups, the reduction in stress would be greater than when done individually. This hypothesis is consistent with the stress-buffering social support hypothesis, which suggests that feelings of connection and support mitigate experiences of stress (Cohen and Wills, 1985). However, both group and individual chanting conferred similar decreases in stress. One interpretation of this finding is that the reduction of stress observed following chanting resulted from the changes in breathing and heart rate arising from the physical act of chanting, and not as a secondary consequence of the connection and support that may have been experienced during group chanting.

However, regression modeling suggested that the impact of chanting on stress reduction may depend on other factors. First, individuals with high engagement in the intervention experienced greater stress reduction than those with low engagement. Given the cognitive and physical demands of chanting, high levels of engagement may optimize the potential benefits of chanting. Second, individuals with high trait anxiety are more likely than individuals with low trait anxiety to experience stress reduction, presumably because those with low levels of anxiety have little need or scope to reduce their stress levels. Third, stress reduction was most evident for individuals with greater chanting experience, suggesting that experienced chanters are skilled at reducing their stress levels.

### Mood

There was a significant increase in positive affect for participants in the chanting conditions, compared to a slight decrease in positive affect for participants in the control conditions. However, contrary to expectations, both group and individual chanting conditions led to similar increases in positive affect. This finding suggests that the physical act of chanting may lead to positive affect rather than from feelings of connection that arise from chanting in a group.

There was no significant difference in negative affect between chanting and control groups, or between group and individual chanting conditions. As shown in Table 1, however, negative affect decreased for all conditions. Regression modeling revealed two predictors of this decrease in negative affect. First, individuals with high trait anxiety were most likely to experience a decrease in negative affect following the interventions (whether chanting or listening). Second, negative affect decreased more for participants with high levels of engagement. Given all conditions involved attending closely to the intervention (chanting or listening), it is possible that focused attention led to a decrease in negative cognitions in all conditions. Rumination on negative thoughts should reinforce negative mood states, whereas engaging strongly on an intervention should distract participants from negative cognitions (Nolen-Hoeksema et al., 2008). Thus, one reason that negative affect was reduced across conditions is that the attention directed toward the interventions inhibited ruminative thinking to a comparable degree.

### Group Connection

Participants in the two online group conditions were assessed for feelings of connectedness to others in their group. As hypothesized, participants in the group chanting condition experienced significantly higher levels of connection to the group than the control condition; participants in the group chanting condition reported increased feelings of connection after chanting, whereas participants in the group control condition reported a minor decrease in connection after the listening task.

### General Connection

Online chanting had no significant effect on broader feelings of connection to all people, in spite of popular claims that such rituals can lead to a sense of “oneness with the Universe.” That is, chanting increased feelings of connectedness to “in-group” members, but not to people outside their group. This finding represents the first evidence of a dissociation between feelings of connectedness to group members and feelings of connectedness to people in general.

Interestingly, Reddish et al. (2014) reported that explicit synchronization with others can lead to an increase in prosocial behaviors toward both in-group and out-group members. After participants synchronized with one individual in their group, they were likely to display prosocial behavior to individuals outside of their group. This effect was not observed when behaviors were asynchronous in the in-group. The current results do not align with this finding, possibly because the close synchronization that was achieved in their study was not achieved in our online group chanting intervention. In the study by Reddish et al. (2014), participants synchronized with one another explicitly and precisely. In the current investigation, participants in the group chanting condition synchronized with an audio recording of chanting, and only secondarily with one another. Quite possibly, feelings of connection to others may have been stronger if participants had explicitly synchronized with other people, rather than with an audio recording of chanting.

### Challenges and Future Prospects

There are inherent and unavoidable challenges in studying online group and individual chanting. One limitation is we only examined a brief intervention of 10 min of chanting, which is a form of chanting meditation. However, the benefits of meditation are known to be dose-dependent (Tang et al., 2015), and only one dosage was examined in our investigation. Thus, future research should examine the effects of chanting over an extended period of time to assess the dose-dependence of chanting for changes in stress, mood, and group connection.

A second limitation of the investigation was the reliance on self-report measures. Given the online nature of our investigation, and the pandemic-related restrictions on investigations involving human participation, it was not feasible to measure biological indicators of stress such as heart rate variability or cortisol levels. Once pandemic restrictions are lifted, future research should include biological indicators of stress following online chanting interventions, which may corroborate the self-report results in this investigation, and shed light on biological mechanisms.

A third limitation is that our group and individual chanting conditions differed in more than just group size. Specifically, the group chanting condition included an audio recording, whereas the individual chanting condition did not. A recording was included in the group chanting condition because existing technologies are non-optimal for livestream coordinated group chanting, whereby variable delays in auditory feedback result in asynchrony among participants. The audio-recording ensured that participants had a common signal to pace their chanting, and simulated the auditory feedback that would normally be present in a group chanting activity. For the individual chanting condition, no audio recording was included to ensure that participants felt alone, given that the presence of an audio recording might imply to participants that they were chanting with another individual. These differences also meant that participants in the group condition breathed according to a pacing signal, whereas participants in the individual condition chanted (and hence breathed) at their own natural pace. Such differences may have introduced uncontrolled variability in responding, which could explain why we did not observe reliable differences between individual and group conditions. With technological advances, it may be possible in future research to omit the recording, and instruct participants in the group condition to consciously synchronize their collective chanting. Other methodological adjustments could be made to ensure that breathing and chanting rates are comparable in group and individual conditions. Such refinements may reduce variability in responding, and help to isolate differences in the impact of individual and group chanting.

The current investigation considered a number of psychosocial measures based on an assessment of effects observed in previous relevant research. Future research should include other dependent measures such as prosocial behavior, and other independent variables such as gender. Notably, gender was not a significant predictor of outcomes in our models, but it seems possible that gender may be relevant to other effects of chanting. It would therefore be valuable to replicate this study with a larger sample size and measures for which gender differences may be relevant.

To conclude, this investigation corroborates previous reports that chanting can benefit stress and mood (Bernardi et al., 2001; Wolf and Abell, 2003; Perry et al., 2016), but makes a novel contribution by confirming that such benefits are preserved in an online setting. As we were unable to compare the benefits of online and live chanting interventions, it is unknown whether the benefits of live chanting are attenuated or enhanced in an online setting. Nonetheless, in an era of social distancing, when online formats may be the only viable option, it is important to confirm that online chanting can confer significant benefits, and may be an effective online tool for reducing stress, increasing positive affect, and enhancing feelings of connection.

## Data Availability Statement

The data presented in this study are openly available in [OSF] at doi: 10.17605/OSF.IO/WE2XN.

## Ethics Statement

The studies involving human participants were reviewed and approved by Human Sciences Ethics Committee, Macquarie University, Reference No: 52020637515113. The patients/participants provided their written informed consent to participate in this study.

## Author Contributions

All authors contributed to the design, analysis, and interpretation of these data. Felicity Simpson managed recruitment and testing. All authors contributed to the final manuscript.

## Conflict of Interest

The authors declare that the research was conducted in the absence of any commercial or financial relationships that could be construed as a potential conflict of interest.

## Appendix: Pre- and Post-Scores of Outcome Measures

**TABLE A1:**

		Chanting individual	Chanting group	Control individual	Control group
Pre-intervention stress	M	33.25	34.552	35.179	33.5
	SD	7.988	9.833	8.349	9.172
Post-intervention stress	M	29.179	30.483	34.893	32.906
	SD	9.50	10.391	8.381	10.590
Pre-intervention positive affect	M	33	27.759	30.964	34.188
	SD	9.537	9.452	8.14	8.789
Post-intervention positive affect	M	34.321	30.897	28.357	30.469
	SD	10.670	9.416	8.795	11.066
Pre-intervention negative affect	M	14.036	15.655	15.786	14.094
	SD	5.037	8.528	7.259	4.510
Post-intervention negative affect	M	12.25	13.62	13.464	13.0313
	SD	3.758	6.316	5.897	4.425

**TABLE A2:** Social connectedness (general).

		Chanting individual	Chanting group	Control individual	Control group
Pre-intervention social connectedness (general)	M	15.67857	17.37931	17.42857	16.90625
	SD	4.489111	5.621475	7.041825	7.62576
Post-intervention social connectedness (general)	M	14.75	17.58621	18.75	17.65625
	SD	3.718074	6.026828	8.240033	6.403675

**TABLE A3:** Social connectedness to members in group.

		Chanting group	Control group
Pre-intervention connectedness to group members	M	4	3.9375
	SD	1.752549	1.605183
Post-intervention connectedness to group members	M	4.551724	3.90625
	SD	1.702649	1.747983
